# Supplementary material for: Transition cow health and management in pasture-based dairy herds: A farmers’ survey
Source: PLoS One. 2024 Dec 17;19(12):e0314987. doi: 10.1371/journal.pone.0314987 (PMC11651598; doi:10.1371/journal.pone.0314987)
Supplement: S1 Table — The survey was made using Survey Monkey (SurveyMonkey Inc., Palo Alto, CA) but has been presented here in table format. (DOCX) [file pone.0314987.s001.docx]

**S1 Table**

| Question | Response options |
| --- | --- |
| Q1. Overall, in which stage of lactation do you see the highest incidence of disease? | - Fresh calver (first 3 weeks after calving) |
|  | - Early lactation (from week 3 to end of 3rd month of lactation) |
|  | - Mid lactation (from start of 4th month to end of 7th month of lactation) |
|  | - Late lactation (from start of 8th month of lactation to dry-off) |
|  | - Far-off dry (from dry-off to close-up) |
|  | - Close-up dry (last 3 weeks of pregnancy) |
|  | |
| Q2. When in the calving season do you see the highest incidence of disease? | - At the beginning (with early calvers) |
|  | - At the end (with late calvers) |
|  | - Problems arise during all the calving season regardless of the stage |
|  | |
| Q3. In which cows do you see the highest incidence of disease during the calving season? | - Primiparous (first time calvers) |
|  | - Multiparous (second and greater time calvers) |
|  | - Disease affects both, primiparous and multiparous cows |
|  | |
| Q4. Please select the appropriate option regarding the following conditions in your herd: acidosis, displaced abomasum (LDA, RDA) and/or digestive problems, dystocia, fatty liver, grass tetany, ketosis, mastitis, metritis (wash out, dirty cow), milk fever and/or downer cow, retained placenta (held cleaning), subclinical hypocalcaemia (subclinical milk fever), subclinical ketosis | - Significant problem (regularly treating severe cases with some cows lost/culled) - Routine problem (regularly treating cows to control issues) - Occasional cases but no major effect on herd performance - Not a problem for my herd - I don’t know if it is a problem for my herd. |
|  |  |
| Q5. What % of cows are treated on an 'average' year on your farm for: displaced abomasum (LDA, RDA) and/or digestive problems, grass tetany, ketosis, mastitis, metritis, milk fever and/or downer cow, retained placenta (held cleaning) | - <1% |
|  | - 1-3% |
|  | - 4-6% |
|  | - 6-10% |
|  | - >10% |
|  | |
| Q6. Please indicate which type of records do you keep in association with the following conditions: displaced abomasum (LDA, RDA) and/or digestive problems, grass tetany, ketosis, mastitis, metritis, milk fever and/or downer cow, retained placenta (held cleaning) | - Incidence (affected cows) |
|  | - Antibiotic treatment |
|  | - Supportive treatment (no antibiotic) |
|  | - I don’t keep records of this disease |
|  | |
| Q7. Do you implement any of the following management strategies for dry cows? Please select all that apply | - Body condition monitoring during the dry period |
|  | - Management in >1 group (e.g. separate groups for fat and thin cows) |
|  | - Acidifying diet to close-up dry (late pregnancy) cows (DCAD diets) |
|  | - Low potassium diet to dry cows (lower potassium silage for dry cows) |
|  | - Calcium supplementation to close-up dry (late pregnancy) cows' diet |
|  | - Magnesium supplementation to close-up dry (late pregnancy) cows' diet |
|  | - Vitamin D supplementation to close-up dry (late pregnancy) cows' diet |
|  | - Provide feed sources other than silage to close-up dry (late pregnancy) cows (e.g. concentrates, grains, - non-forage supplements) |
|  | - Other (please specify) |
|  | |
| Q8. Do you implement any of the following management strategies for freshly calved  cows? Please select all that apply | - Once a day milking for a few days after calving (in early lactation) |
|  | - Keep freshly calved cows indoors for a period after calving |
|  | - All cows routine calcium supplementation (e.g. bottle, bolus) at calving |
|  | - High-risk cows calcium supplementation (e.g. bottle, bolus) at calving |
|  | - Calcium supplementation to freshly calved cows' diet |
|  | - Magnesium supplementation to freshly calved cows' diet |
|  | - Vitamin D supplementation to freshly calved cows' diet |
|  | - Other (please specify) |
|  | |
| Q9. Do you get advice from any of the following professionals regarding the management of your cows over the dry and freshly calved periods? Please select all that apply | - Yes, from my Teagasc advisor |
|  | - Yes, from my nutritionist |
|  | - Yes, from my veterinarian |
|  | - Yes, from sales representatives |
|  | - No, I don't get advice from any of the above |
|  | |
| Q10. Please select the option that best describes your herd calving pattern | - Spring-calving only |
|  | - Autumn calving only |
|  | - Split calving |
|  | - All year round calving |
|  | |
| Q11. Please select the option that best describes your herd feeding strategy | - Grazing high-input (>1 tone of bought-in feed/cow) |
|  | - Grazing low-input (≤1 tone of bought-in feed/cow) |
|  | - Zero grazed grass all time |
