# Supplementary material for: Transition cow health and management in pasture-based dairy herds: A farmers’ survey
Source: PLoS One. 2024 Dec 17;19(12):e0314987. doi: 10.1371/journal.pone.0314987 (PMC11651598; doi:10.1371/journal.pone.0314987)
Supplement: S2 Table — aHerds were categorized by herd size (large: >150 cows, above average: 100–150 cows, average: 60–100 cows, or small: <60 cows) using the Irish national dairy herd average as reference (93 cows; [9]), and by calving pattern (spring-calving: cows calving in spring, or split-calving: cows calving in spring and autumn). bIQR = Interquartile range. (DOCX) [file pone.0314987.s002.docx]

**S2 Table**

|  | Herd size^a^ | | | |  | Herd calving pattern^a^ | |  |
| --- | --- | --- | --- | --- | --- | --- | --- | --- |
| Items | Large | Above average | Average | Small |  | Spring-calving | Split-calving | All |
| Herd size | n = 154 | n = 134 | n = 148 | n = 74 |  | n = 436 | n = 67 | n = 510 |
| Median herd size, n cows | 204 | 120 | 82 | 49 |  | 109 | 135 | 110 |
| Herd size IQR^b^, n cows | 169-284 | 110-132 | 72-91 | 38-56 |  | 75-156 | 95-216 | 78-162 |
| Milk yield | n = 71 | n = 52 | n = 75 | n = 39 |  | n = 214 | n = 20 | n = 237 |
| Mean 305-day milk yield, L | 6,526 | 6,783 | 6,697 | 7,865 |  | 6,818 | 7,293 | 6,857 |
| 305-day milk yield IQR, L | 5,935-6,965 | 6,418-7,131 | 6,101-7,262 | 5,774-7,279 |  | 6,091-7,084 | 6,825-7,768 | 6,111 - 7,162 |
| Calving interval | n = 107 | n = 76 | n = 95 | n = 45 |  | n = 267 | n = 45 | n = 323 |
| Mean calving interval, days | 376 | 379 | 374 | 378 |  | 372 | 398 | 377 |
| Calving interval IQR, days | 367-383 | 367-383 | 367-377 | 367-379 |  | 366-375 | 384-406 | 367-381 |
| Bought-in feed per cow | n = 154 | n = 134 | n = 144 | n = 73 |  | n = 436 | n = 67 | n = 519 |
| >1 tonne, % | 44.2 | 59.0 | 54.9 | 46.6 |  | 46.8 | 77.6 | 51.6 |
| <1 tonne, % | 54.6 | 41.0 | 44.4 | 53.4 |  | 53.2 | 19.4 | 47.8 |
| Zero-grazed grass fed all of the time, % | 1.3 | 0.0 | 0.7 | 0.0 |  | 0.0 | 3.0 | 0.6 |
