# Supplementary material for: Transition cow health and management in pasture-based dairy herds: A farmers’ survey
Source: PLoS One. 2024 Dec 17;19(12):e0314987. doi: 10.1371/journal.pone.0314987 (PMC11651598; doi:10.1371/journal.pone.0314987)
Supplement: S3 Table — aHerds were categorized by herd size (large: >150 cows, above average: 100–150 cows, average: 60–100 cows, or small: <60 cows) using the Irish national dairy herd average as reference (93 cows; [9]), and by calving pattern (spring-calving: cows calving in spring, or split-calving: cows calving in spring and autumn). bStages of lactation: Fresh calver: First 3 weeks after calving, early lactation: from week 3 to end of 3rd month of lactation, mid lactation: from start of 4th month to end of 7th month of lactation, late lactation: from start of 8th month of lactation to dry-off, far-off dry: from dry-off to close-up, close-up dry: last 3 weeks of pregnancy. (DOCX) [file pone.0314987.s003.docx]

**S3 Table**

|  | Herd size^a^ | | | |  | Herd calving pattern^a^ | |  |
| --- | --- | --- | --- | --- | --- | --- | --- | --- |
|  | Large | Above average | Average | Small |  | Spring-calving | Split-calving | All |
| Parity | n = 154 | n = 131 | n = 145 | n = 69 |  | n = 431 | n = 65 | n = 513 |
| Multiparous | 56.5 | 47.3 | 49.7 | 52.2 |  | 52.4 | 53.9 | 51.9 |
| Primiparous | 5.2 | 5.3 | 6.2 | 2.9 |  | 5.3 | 1.5 | 5.1 |
| All parities | 38.3 | 47.3 | 44.1 | 44.9 |  | 42.2 | 44.6 | 43.1 |
| Stage of lactation^b^ | n = 154 | n = 132 | n = 146 | n = 73 |  | n = 437 | n = 65 | n = 519 |
| Fresh calver | 66.2 | 62.1 | 45.9 | 53.4 |  | 57.0 | 60.0 | 57.6 |
| Early lactation | 26.0 | 28.8 | 35.6 | 26.0 |  | 29.8 | 27.7 | 29.7 |
| Mid lactation | 2.0 | 3.0 | 6.9 | 6.9 |  | 5.0 | 1.5 | 4.6 |
| Late lactation | 4.6 | 4.6 | 10.3 | 9.6 |  | 6.4 | 10.8 | 6.9 |
| Far-off dry | 0.7 | 0.8 | 0.7 | 4.1 |  | 1.4 | 0.0 | 1.2 |
| Close-up dry | 0.7 | 0.8 | 0.7 | 0.0 |  | 0.5 | 0.0 | 0.6 |
| Calving season stage | n = 152 | n = 129 | n = 143 | n = 72 |  | n = 427 | n = 66 | n = 510 |
| Beginning | 10.5 | 9.3 | 10.5 | 13.9 |  | 10.5 | 12.1 | 10.6 |
| End | 51.3 | 45.0 | 51.8 | 41.7 |  | 47.1 | 53.0 | 48.0 |
| All | 38.2 | 45.7 | 37.8 | 44.4 |  | 42.4 | 34.9 | 41.4 |
