# Supplementary material for: Transition cow health and management in pasture-based dairy herds: A farmers’ survey
Source: PLoS One. 2024 Dec 17;19(12):e0314987. doi: 10.1371/journal.pone.0314987 (PMC11651598; doi:10.1371/journal.pone.0314987)
Supplement: S4 Table — Perception was based on treatments, mortality, culling and herd performance (% of respondents to a transition period survey in Ireland). aHerds were categorized by herd size (large: >150 cows, above average: 100–150 cows, average: 60–100 cows, or small: <60 cows) using the Irish national dairy herd average as reference (93 cows; [9]), and by calving pattern (spring-calving: cows calving in spring, or split-calving: cows calving in spring and autumn). bPerception definitions: Significant problem (regularly treating severe cases with some cows lost/culled), routine problem (regularly treating cows to control issues), occasional cases (but no major effect on herd performance). (DOCX) [file pone.0314987.s004.docx]

**S4 Table**

|  | Herd size^a^ | | | |  | Herd calving pattern^a^ | |  |
| --- | --- | --- | --- | --- | --- | --- | --- | --- |
| Condition and perception^b^ | Large | Above average | Average | Small |  | Spring-calving | Split-calving | All |
| Milk fever and/or downer cow | n = 154 | n = 133 | n = 147 | n = 74 |  | n = 438 | n = 67 | n = 522 |
| Significant problem | 1.3 | 3.0 | 2.0 | 2.7 |  | 2.7 | 0.0 | 2.3 |
| Routine problem | 18.8 | 14.3 | 7.5 | 10.8 |  | 13.2 | 13.4 | 13.4 |
| Occasional cases | 69.5 | 70.7 | 77.6 | 77.0 |  | 72.6 | 76.1 | 73.0 |
| Not a problem | 10.4 | 12.0 | 12.9 | 9.5 |  | 11.4 | 10.5 | 11.3 |
| I don't know | 0.0 | 0.0 | 0.0 | 0.0 |  | 0.0 | 0.0 | 0.0 |
| Subclinical hypocalcaemia | n = 154 | n = 133 | n = 147 | n = 74 |  | n = 437 | n = 67 | n = 522 |
| Significant problem | 0.7 | 0.0 | 1.4 | 1.4 |  | 1.1 | 0.0 | 1.0 |
| Routine problem | 13.6 | 6.8 | 6.1 | 5.4 |  | 8.5 | 9.0 | 8.4 |
| Occasional cases | 48.1 | 48.1 | 42.2 | 35.1 |  | 44.4 | 44.8 | 44.4 |
| Not a problem | 23.4 | 21.8 | 28.6 | 32.4 |  | 24.7 | 28.4 | 25.5 |
| I don't know | 14.3 | 23.3 | 21.8 | 25.7 |  | 21.3 | 17.9 | 20.7 |
| Retained placenta | n = 153 | n = 131 | n = 146 | n = 73 |  | n = 433 | n = 67 | n = 517 |
| Significant problem | 0.7 | 0.8 | 0.7 | 0.0 |  | 0.7 | 0.0 | 0.6 |
| Routine problem | 7.8 | 2.3 | 4.8 | 5.5 |  | 5.3 | 4.5 | 5.0 |
| Occasional cases | 66.0 | 70.2 | 68.5 | 71.2 |  | 67.9 | 74.6 | 69.1 |
| Not a problem | 25.5 | 26.7 | 25.3 | 23.3 |  | 25.9 | 20.9 | 25.2 |
| I don't know | 0.0 | 0.0 | 0.7 | 0.0 |  | 0.2 | 0.0 | 0.2 |
| Metritis | n = 153 | n = 131 | n = 147 | n = 74 |  | n = 434 | n = 67 | n = 518 |
| Significant problem | 0.7 | 0.0 | 0.7 | 0.0 |  | 0.5 | 0.0 | 0.4 |
| Routine problem | 11.1 | 5.3 | 4.8 | 5.4 |  | 7.6 | 6.0 | 7.1 |
| Occasional cases | 71.9 | 74.8 | 75.5 | 62.2 |  | 70.5 | 80.6 | 72.2 |
| Not a problem | 16.3 | 20.0 | 19.1 | 31.1 |  | 21.2 | 13.4 | 20.1 |
| I don't know | 0.0 | 0.0 | 0.0 | 1.4 |  | 0.2 | 0.0 | 0.2 |
| Acidosis | n = 153 | n = 134 | n = 147 | n = 74 |  | n = 436 | n = 67 | n = 521 |
| Significant problem | 0.0 | 0.8 | 0.0 | 1.4 |  | 0.5 | 0.0 | 0.4 |
| Routine problem | 2.0 | 2.2 | 1.4 | 2.7 |  | 2.1 | 0.0 | 1.9 |
| Occasional cases | 45.8 | 34.3 | 44.9 | 35.1 |  | 42.0 | 43.3 | 41.8 |
| Not a problem | 44.4 | 56.7 | 45.6 | 47.3 |  | 48.0 | 44.8 | 47.6 |
| I don't know | 7.8 | 6.0 | 8.2 | 13.5 |  | 7.6 | 11.9 | 8.3 |
| Displaced abomasum | n = 153 | n = 134 | n = 147 | n = 74 |  | n = 437 | n = 67 | n = 522 |
| Significant problem | 0.7 | 0.0 | 0.0 | 1.4 |  | 0.5 | 0.0 | 0.4 |
| Routine problem | 0.7 | 4.5 | 2.0 | 1.4 |  | 2.1 | 3.0 | 2.3 |
| Occasional cases | 67.3 | 66.4 | 58.5 | 51.4 |  | 62.2 | 62.7 | 61.9 |
| Not a problem | 30.7 | 29.1 | 38.1 | 43.2 |  | 34.1 | 34.3 | 34.5 |
| I don't know | 0.7 | 0.0 | 1.4 | 2.7 |  | 1.1 | 0.0 | 1.0 |
| Grass tetany | n = 154 | n = 133 | n = 147 | n = 73 |  | n = 436 | n = 67 | n = 521 |
| Significant problem | 0.7 | 0.0 | 0.7 | 0.0 |  | 0.2 | 1.5 | 0.4 |
| Routine problem | 1.3 | 0.0 | 0.7 | 6.9 |  | 1.4 | 1.5 | 1.5 |
| Occasional cases | 42.2 | 39.1 | 28.6 | 35.6 |  | 38.5 | 32.8 | 37.2 |
| Not a problem | 55.8 | 59.4 | 68.7 | 57.5 |  | 59.2 | 64.2 | 60.1 |
| I don't know | 0.0 | 1.5 | 1.4 | 0.0 |  | 0.7 | 0.0 | 0.8 |
| Dystocia | n = 150 | n = 134 | n = 147 | n = 74 |  | n = 437 | n = 64 | n = 519 |
| Significant problem | 0.0 | 0.0 | 0.0 | 0.0 |  | 0.0 | 0.0 | 0.0 |
| Routine problem | 0.7 | 0.0 | 0.0 | 1.4 |  | 0.5 | 0.0 | 0.4 |
| Occasional cases | 16.7 | 17.2 | 15.7 | 12.2 |  | 16.9 | 15.6 | 16.2 |
| Not a problem | 47.3 | 45.5 | 51.7 | 54.1 |  | 48.5 | 45.3 | 48.8 |
| I don't know | 35.3 | 37.3 | 32.7 | 32.4 |  | 34.1 | 39.1 | 34.7 |
| Fatty liver | n = 153 | n = 133 | n = 146 | n = 74 |  | n = 435 | n = 67 | n = 520 |
| Significant problem | 0 | 0 | 0 | 0 |  | 0 | 0 | 0 |
| Routine problem | 0.7 | 0 | 0 | 0 |  | 0.2 | 0 | 0.2 |
| Occasional cases | 9.8 | 3.8 | 6.9 | 12.2 |  | 7.8 | 9 | 8.3 |
| Not a problem | 73.2 | 68.4 | 73.3 | 62.2 |  | 70.1 | 74.6 | 70.2 |
| I don't know | 16.3 | 27.8 | 19.9 | 25.7 |  | 21.8 | 16.4 | 21.4 |
| Ketosis | n = 154 | n = 134 | n = 147 | n = 74 |  | n = 438 | n = 67 | n = 523 |
| Significant problem | 0.0 | 0.0 | 0.0 | 0.0 |  | 0.0 | 0.0 | 0.0 |
| Routine problem | 3.9 | 0.0 | 2.0 | 4.1 |  | 2.5 | 1.5 | 2.5 |
| Occasional cases | 60.4 | 62.7 | 62.6 | 54.1 |  | 60.3 | 67.2 | 70.0 |
| Not a problem | 32.5 | 27.6 | 27.9 | 32.4 |  | 31.1 | 22.4 | 29.6 |
| I don't know | 3.3 | 9.7 | 7.5 | 9.5 |  | 6.2 | 9.0 | 7.0 |
| Subclinical ketosis | n = 152 | n = 131 | n = 147 | n = 72 |  | n = 433 | n = 66 | n = 516 |
| Significant problem | 0.0 | 0.0 | 0.0 | 0.0 |  | 0.0 | 0.0 | 0.0 |
| Routine problem | 2.0 | 0.0 | 0.7 | 1.4 |  | 0.9 | 3.0 | 1.2 |
| Occasional cases | 32.2 | 24.4 | 29.9 | 23.6 |  | 27.9 | 34.9 | 28.9 |
| Not a problem | 40.8 | 42.0 | 38.1 | 38.9 |  | 39.3 | 37.9 | 39.5 |
| I don't know | 25.0 | 33.6 | 31.3 | 36.1 |  | 31.9 | 24.2 | 30.4 |
