# Supplementary material for: Transition cow health and management in pasture-based dairy herds: A farmers’ survey
Source: PLoS One. 2024 Dec 17;19(12):e0314987. doi: 10.1371/journal.pone.0314987 (PMC11651598; doi:10.1371/journal.pone.0314987)
Supplement: S5 Table — aHerds were categorized by herd size (large: >150 cows, above average: 100–150 cows, average: 60–100 cows, or small: <60 cows) using the Irish national dairy herd average as reference (93 cows; [9]), and by calving pattern (spring-calving: cows calving in spring, or split-calving: cows calving in spring and autumn). bDCAD = Dietary cation anion difference. (DOCX) [file pone.0314987.s005.docx]

**S5 Table**

|  | Herd size^a^ | | | |  | Herd calving pattern^a^ | |  |
| --- | --- | --- | --- | --- | --- | --- | --- | --- |
| Dry period management strategy | Large | Above average | Average | Small |  | Spring-calving | Split-calving | All |
| Respondents | n = 148 | n = 129 | n = 142 | n = 72 |  | n = 428 | n = 67 | n = 497 |
| Body condition monitoring | 75.0 | 69.0 | 72.5 | 73.6 |  | 73.1 | 61.2 | 73.4 |
| Mg and/or dry cow mineral supplement in diet | 66.9 | 53.5 | 59.2 | 58.3 |  | 60.5 | 55.2 | 61.2 |
| Management in >1 group | 64.9 | 53.5 | 57.0 | 27.8 |  | 57.0 | 38.8 | 55.1 |
| Provide feed sources other than silage | 48.0 | 41.1 | 49.3 | 48.6 |  | 43.0 | 64.2 | 47.1 |
| Calcium supplementation in diet | 35.8 | 39.5 | 25.4 | 37.5 |  | 33.6 | 34.3 | 34.8 |
| Vitamin D supplementation | 20.9 | 22.5 | 23.9 | 19.4 |  | 22.0 | 22.4 | 22.7 |
| Low potassium diet | 25.0 | 20.2 | 21.1 | 9.7 |  | 19.2 | 22.4 | 20.3 |
| Negative DCAD^b^ diet | 10.1 | 3.9 | 3.5 | 8.3 |  | 5.4 | 9.0 | 6.2 |
