# Supplementary material for: Transition cow health and management in pasture-based dairy herds: A farmers’ survey
Source: PLoS One. 2024 Dec 17;19(12):e0314987. doi: 10.1371/journal.pone.0314987 (PMC11651598; doi:10.1371/journal.pone.0314987)
Supplement: S6 Table — aHerds were categorized by herd size (large: >150 cows, above average: 100–150 cows, average: 60–100 cows, or small: <60 cows) using the Irish national dairy herd average as reference (93 cows; [9]), and by calving pattern (spring-calving: cows calving in spring, or split-calving: cows calving in spring and autumn). (DOCX) [file pone.0314987.s006.docx]

**S6 Table**

|  | Herd size^a^ | | | |  | Herd calving pattern^a^ | |  |
| --- | --- | --- | --- | --- | --- | --- | --- | --- |
| Fresh period management strategy | Large | Above average | Average | Small |  | Spring-calving | Split-calving | All |
| Respondents | n = 148 | n = 121 | n = 136 | n = 67 |  | n = 416 | n = 67 | n = 487 |
| Keep cows indoors for a period after calving | 68.2 | 67.8 | 69.1 | 64.2 |  | 71.6 | 41.8 | 68.0 |
| Ca supplementation at calving | 62.2 | 66.1 | 57.4 | 50.7 |  | 58.2 | 68.7 | 60.6 |
| Once-a-day milking for a few days after calving | 43.2 | 38.8 | 30.9 | 26.9 |  | 39.9 | 9.0 | 35.9 |
| Mg and/or mineral supplement in diet | 32.4 | 28.1 | 25.0 | 26.9 |  | 28.4 | 22.4 | 28.3 |
| Ca supplementation in diet | 17.6 | 22.3 | 11.8 | 23.9 |  | 17.3 | 16.4 | 18.1 |
| Vitamin D supplementation | 12.2 | 5.8 | 8.1 | 11.9 |  | 9.6 | 7.5 | 9.7 |
