# Supplementary material for: Transition cow health and management in pasture-based dairy herds: A farmers’ survey
Source: PLoS One. 2024 Dec 17;19(12):e0314987. doi: 10.1371/journal.pone.0314987 (PMC11651598; doi:10.1371/journal.pone.0314987)
Supplement: Attachment — Submitted filename: PONE-D-24-31981_reviewer.pdf [file pone.0314987.s007.pdf]

PLOS ONE

Transition cow health and management in pasture-based dairy herds: a farmers' survey

--Manuscript Draft--

**Manuscript Number:**	PONE-D-24-31981
**Article Type:**	Research Article
**Full Title:**	Transition cow health and management in pasture-based dairy herds: a farmers' survey
**Short Title:**	Transition cow health and management in pasture-based dairy herds: a farmers' survey
**Corresponding Author:**	Ainhoa Valldecabres, Ph.D.<br>Teagasc Animal and Grassland Research and Innovation Centre<br>Fermoy, IRELAND
**Keywords:**	Fresh period; dry period; disease incidence; milk fever; management strategies; cow; dairy cow; grazing cow; hypocalcemia; subclinical hypocalcemia
**Abstract:**	Seasonal-calving pasture-based systems characterize Irish dairy production. Compared to other grazing systems, dry cows are housed and offered a diet dominated by grass silage, providing unique opportunities for transition cow management. This study aimed to describe transition period disease incidence and management strategies reported by farmers and to evaluate their associations with herd size and calving pattern to inform and guide research activities and national advisory. An online survey distributed amongst 3,899 Teagasc Technical Dairy Advisory clients yielded 525 responses suitable for analysis. Disease incidence was reportedly highest in cows within 3 weeks post-calving (58%), at the end of the calving season (48%) and multiparous cows (52%). Twenty-three percent of respondents reported to treat >3% of their herd for milk fever. Regarding transition cow management, dry cow body condition monitoring (73%), dry cow mineral supplementation (61%), and Ca supplementation at calving (61%) were the most commonly reported. Other dry cow management strategies for milk fever prevention supported by research in other production systems were not commonly reported (low K [20%] and negative dietary cation-anion difference diet [6%]). Compared to spring-calving, the odds of reporting to provide feeds besides grass silage to dry cows were higher (OR = 2.5) while the odds of reporting to implement once-a-day milking (OR = 0.16) were lower for farmers from split-calving herds. The odds of reporting to keep records of antibiotic treatment for milk fever were higher (OR = 3.20) for farmers from small compared to large herds. In conclusion, our results suggest that milk fever is a transition cow health concern in Irish dairy farms. Efforts should be devoted to enhance farmers' uptake of existing research-supported prophylactic strategies for milk fever and to optimize commonly reported management strategies in the Irish dairy production context.
**Order of Authors:**	Louise Horan
	Joseph Patton
	Conor G McAloon
	Ángel García-Muñoz
	Áine Regan
	John F Mee
	Ainhoa Valldecabres, Ph.D.
**Additional Information:**	
**Question**	**Response**
**Financial Disclosure**	The author(s) received no specific funding for this work.

Powered by Editorial Manager® and ProduXion Manager® from Aries Systems Corporation

Enter a financial disclosure statement that describes the sources of funding for the work included in this submission. Review the submission guidelines for detailed requirements. View published research articles from *PLOS ONE* for specific examples.<br><br>This statement is required for submission and **will appear in the published article** if the submission is accepted. Please make sure it is accurate.<br><br>**Funded studies**<br>Enter a statement with the following details:<br>• Initials of the authors who received each award<br>• Grant numbers awarded to each author<br>• The full name of each funder<br>• URL of each funder website<br>• Did the sponsors or funders play any role in the study design, data collection and analysis, decision to publish, or preparation of the manuscript?<br><br>Did you receive funding for this work?	
**Competing Interests**<br><br>Use the instructions below to enter a competing interest statement for this submission. On behalf of all authors, disclose any competing interests that could be perceived to bias this work—acknowledging all financial support and any other relevant financial or non-financial competing interests.<br><br>This statement is required for submission and **will appear in the published article** if the submission is accepted. Please make sure it is accurate and that any funding sources listed in your Funding Information later in the submission form are also declared in your Financial Disclosure statement.<br><br>View published research articles from	The authors have declared that no competing interests exist.

PLOS ONE for specific examples.<br><br>**NO authors have competing interests**<br><br>Enter: *The authors have declared that no competing interests exist.*<br><br>**Authors with competing interests**<br><br>Enter competing interest details beginning with this statement:<br><br>*I have read the journal's policy and the authors of this manuscript have the following competing interests: [insert competing interests here]*<br><br>* typeset	
**Ethics Statement**<br><br>Enter an ethics statement for this submission. This statement is required if the study involved:<br><br>• Human participants<br>• Human specimens or tissue<br>• Vertebrate animals or cephalopods<br>• Vertebrate embryos or tissues<br>• Field research<br><br>Write "N/A" if the submission does not require an ethics statement.<br><br>General guidance is provided below. Consult the submission guidelines for detailed instructions. **Make sure that all information entered here is included in the Methods section of the manuscript.**	The present study was approved by the University College Dublin Human Research Ethics Committee – Sciences (LS-LR-22-180; HREC-LS). A tick the box question at the beginning of the survey was used to obtain written consent from respondents to use data provided in the survey and that available in their ICBF profiles for the purpose of this study.<br>Animals were not used in the present study. Therefore, no ethical approval regarding the use of animals was sought in this study.

Powered by Editorial Manager® and ProduXion Manager® from Aries Systems Corporation

**Format for specific study types**<br><br>**Human Subject Research (involving human participants and/or tissue)**<br>• Give the name of the institutional review board or ethics committee that approved the study<br>• Include the approval number and/or a statement indicating approval of this research<br>• Indicate the form of consent obtained (written/oral) or the reason that consent was not obtained (e.g. the data were analyzed anonymously)<br><br>**Animal Research (involving vertebrate animals, embryos or tissues)**<br>• Provide the name of the Institutional Animal Care and Use Committee (IACUC) or other relevant ethics board that reviewed the study protocol, and indicate whether they approved this research or granted a formal waiver of ethical approval<br>• Include an approval number if one was obtained<br>• If the study involved *non-human primates*, add *additional details* about animal welfare and steps taken to ameliorate suffering<br>• If anesthesia, euthanasia, or any kind of animal sacrifice is part of the study, include briefly which substances and/or methods were applied<br><br>**Field Research**<br><br>Include the following details if this study involves the collection of plant, animal, or other materials from a natural setting:<br>• Field permit number<br>• Name of the institution or relevant body that granted permission	
**Data Availability**<br><br>Authors are required to make all data underlying the findings described fully available, without restriction, and from the time of publication. PLOS allows rare exceptions to address legal and ethical concerns. See the PLOS Data Policy and FAQ for detailed information.	Yes - all data are fully available without restriction

A Data Availability Statement describing where the data can be found is required at submission. Your answers to this question constitute the Data Availability Statement and **will be published in the article**, if accepted.<br><br>**Important:** Stating 'data available on request from the author' is not sufficient. If your data are only available upon request, select 'No' for the first question and explain your exceptional situation in the text box.<br><br>Do the authors confirm that all data underlying the findings described in their manuscript are fully available without restriction?	
**Describe where the data may be found in full sentences. If you are copying our sample text, replace any instances of XXX with the appropriate details.**<br><br>• If the data are **held or will be held in a public repository**, include URLs, accession numbers or DOIs. If this information will only be available after acceptance, indicate this by ticking the box below. For example: *All XXX files are available from the XXX database (accession number(s) XXX, XXX.).*<br>• If the data are all contained **within the manuscript and/or Supporting Information files**, enter the following: *All relevant data are within the manuscript and its Supporting Information files.*<br>• If neither of these applies but you are able to provide **details of access elsewhere**, with or without limitations, please do so. For example:<br><br>*Data cannot be shared publicly because of [XXX]. Data are available from the XXX Institutional Data Access / Ethics Committee (contact via XXX) for researchers who meet the criteria for access to confidential data.*<br><br>*The data underlying the results presented in the study are available from (include the name of the third party*	All relevant data are within the manuscript and its Supporting Information files.

and contact information or URL).<br>• This text is appropriate if the data are owned by a third party and authors do not have permission to share the data.<br><br>* typeset	
Additional data availability information:	

Manuscript Click here to access/download;Manuscript;PlosOne_1.docx

Short title: Pasture-based transition period survey

Transition cow health and management in pasture-based dairy herds: a farmers' survey

Louise Horan[1,2], Joseph Patton[1], Conor G. McAloon[2], Ángel García-Muñoz[3], Áine Regan[4], John F. Mee[1] and, Ainhoa Valldecabres[1*]

[1]Animal and Grassland Research and Innovation Centre, Teagasc Moorepark, Fermoy, Co. Cork, Ireland

[2]School of Veterinary Medicine, University College Dublin, Belfield, Dublin 4, Ireland

[3]Facultad de Veterinaria, Universidad Cardenal Herrera-CEU, CEU Universities, Valencia, Spain

[4]Department of Agri-food Business & Spatial Analysis, Teagasc Mellows Campus, Athenry, Co. Galway, Ireland

[*] Corresponding author

E-mail: Ainhoa.Valldecabres@teagasc.ie (AV)

Abstract

Seasonal-calving pasture-based systems characterize Irish dairy production. Compared to other grazing systems, dry cows are housed and offered a diet dominated by grass silage, providing unique opportunities for transition cow management. This study aimed to describe transition period disease incidence and management strategies reported by farmers and to evaluate their associations with herd size and calving pattern to inform and guide research activities and national advisory. An online survey distributed amongst 3,899 Teagasc Technical Dairy Advisory clients yielded 525 responses suitable for analysis. Disease incidence was reportedly highest in cows within 3 weeks post-calving (58%), at the end of the calving season (48%) and multiparous cows (52%). Twenty-three percent of respondents reported to treat >3% of their herd for milk fever. Regarding transition cow management, dry cow body condition monitoring (73%), dry cow mineral supplementation (61%), and Ca supplementation at calving (61%) were the most commonly reported. Other dry cow management strategies for milk fever prevention supported by research in other production systems were not commonly reported (low K [20%] and negative dietary cation-anion difference diet [6%]). Compared to spring-calving, the odds of reporting to provide feeds besides grass silage to dry cows were higher (OR = 2.5) while the odds of reporting to implement once-a-day milking (OR = 0.16) were lower for farmers from split-calving herds. The odds of reporting to keep records of antibiotic treatment for milk fever were higher (OR = 3.20) for farmers from small compared to large herds. In conclusion, our results suggest that milk fever is a transition cow health concern in Irish dairy farms. Efforts should be devoted to enhance farmers' uptake of existing research-supported prophylactic strategies for milk fever

and to optimize commonly reported management strategies in the Irish dairy production context.

Introduction

The transition period, encompassing the few weeks before and after calving in dairy cows has been a focus of research during the last decades [1–3]. It is not surprising given the range of physical (physiological, immunological and metabolic) and environmental changes which challenge cows' homeostasis and often turn into disease, ultimately impairing cows' welfare and production performance [4–6]. Despite the large amount of research conducted in the transition period and management strategies for its optimization, it remains a challenge to dairy production. The lack of a single definition for the transition period, as well as varying farmers' attitudes towards management and veterinarian involvement have been described as barriers to transition cow health and management improvement by a study involving Canadian farmers and veterinarians [7]. A lack of focussed advice being given to farmers by advisors (subjects of the interviews) due to a perceived lack of interest by the farmer may also restrain the improvement on transition cow health and management as described by Roche [8].

While the challenges faced by housed and grazing cows during the transition period may be similar, system-level differences determine the management possibilities and the occurrence of specific diseases for these two production systems [8,9]. Furthermore, there is wide variability among pasture-based dairy production systems potentially leading to problems unique to each system [8]. In Ireland, dairy herds are predominantly intensive spring-calving herds in which cows graze the majority of their lactational feed requirements and are housed and fed conserved forages during the dry period in the winter months [9,10]. The combination

of grazing and confinement differentiates Ireland's from other pasture-based dairy production systems, and provides unique opportunities for transition cow management. Nevertheless, limited transition cow health research has been conducted in this context and there is a lack of national-level disease incidence and management data which is needed to characterize and benchmark against current scientific recommendations for transition cow health and management strategies implemented in this production system.

Quantitative surveys and cross-sectional observational studies have been used to describe transition cow disease incidence and management strategies in other dairy production systems [10–12]. However, to the best of our knowledge, the only available survey associated with the Irish dairy cow transition period focuses on calving and colostrum management briefly describing pre-calving nutritional management in Irish dairy herds [13]. Therefore, the purpose of this study was to describe farmers' reported disease incidence and management strategies implemented during the transition period, and to quantify their associations with herd size and calving pattern to inform and guide research and advisory activities in transition cow health and management in Irish dairy farms.

Materials and methods

The present study was approved by the University College Dublin Human Research Ethics Committee – Sciences (LS-LR-22-180; HREC-LS). A tick the box question at the beginning of the survey was used to obtain written consent from respondents to use data provided in the survey and that available in their ICBF profiles for the purpose of this study.

Study population

Teagasc Technical Dairy Advisory clients were the target population of this observational study. Teagasc is the Agriculture and Food Development Authority in the Republic of Ireland and is composed of three main pillars: research, education and advisory/extension. Irish farmers voluntarily join the advisory service which aims at disseminating independent, research-driven technical advice and support to clients. This is achieved by means of offering monthly farmer discussion groups, regular on-farm consultations, and provision of decision support packages and printed/audio material. At the time of the study, a total of 3,899 nationwide Irish dairy farmers were members of the Teagasc Technical Dairy Advisory services and had provided consent for being contacted for research purposes; this represents 25.5% of Ireland's dairy farmers in 2022 [14]. The wider dairy farming community could not be targeted in this study due to limitations on personal data access for the researchers.

Survey design and distribution

An online survey was designed to collect information on Irish dairy farmers' transition period perception, disease incidence and implemented management strategies. For the purpose of this study, focus is given to the disease incidence and implemented management strategies survey sections. Questions were modified according to Teagasc dairy advisors' suggestions, and the survey was pilot tested on five people who were either dairy farmers or dairy farm managers to assess its effectiveness and estimate the time to completion. The survey was administered using SurveyMonkey (SurveyMonkey Inc., Palo Alto, CA). At the beginning of the survey the transition period was defined as "late dry (late pregnancy if primiparous) to early lactation period" to provide context to respondents. The survey included 18 questions; questions were a mixture of closed (multiple choice; n = 14), open-ended (n = 3) and multiple

choice with a comment field to allow for providing a not listed response (n = 2). The first question asked respondents to confirm consent to their data from the survey and from their Irish Cattle Breeding Federation (ICBF) profile being used in this study by ticking a box. The second question was for the purpose of data extraction from the ICBF database and was followed by two questions relating to interest on partaking in a subsequent on-farm study. Afterwards, three questions gathered farmers opinions and perception of the transition period, and the remaining questions (n = 11) gathered information to meet the objectives of this study regarding respondent demographics (n = 2), disease incidence (n = 6) and management strategies (n = 4; S1 Table).

The link to the online survey along with an explanatory message were distributed by text message to Teagasc technical dairy advisory clients (n = 3,899) on the 28th September 2022. A reminder text was sent on the 4th October 2022 and the survey was closed for responses 12 days after its opening.

Data processing and analysis

Survey responses were exported to Excel (Excel 2013; Microsoft Corp.) for analysis. Four respondents answered the survey twice; the survey response with the highest level of completion or that provided in the first attempt, if both responses had the same level of completion, were used in the study. Seventy-two respondents skipped every survey question after providing consent for data usage for research purposes and were not included in the analysis. Responses were checked for signs of bot activity before data analysis by checking timestamps to ensure no respondents completed the survey abnormally fast and by checking responses for any illogical or repeated statements [15].

Answers in the open-ended comment fields of some of the multiple choice questions were placed into already existing or new categories within the question for data analysis and summarization. Similarly, some answers to the same question were grouped; given the prevalent inclusion of Mg in pre-made mineral mixes used in Ireland (Reardon et al., unpublished), responses reporting the provision of dry or fresh cow minerals were combined with those reporting Mg supplementation to dry or fresh cows in respective categories named “Mg and/or other mineral supplementation”. And “high-risk cows Ca supplementation at calving” and “all cows routine Ca supplementation at calving” were combined into “Ca supplementation at calving”. Where respondents had the option of selecting an answer or not, the selection of the answer was coded as “yes” and the lack of selection was coded as “no”; consequently, answers such as “I don’t keep records of this disease” and “no, I don't get advice from any of the above” were no longer considered in the analysis as these were already regarded in the above described code. Given the systematic provision of concentrates during milking to lactating cows [16], responses reporting the provision of feeds other than silage to fresh cows were not considered in this study. Answers to reported herd disease treatment incidence were summarized as “above” or “below” previously described herd alarm levels [17]; where the described herd alarm level did not coincide with the answer options specified in the survey, the closest category was referred instead. Only diseases with at least 20% of reported treatments at each side of the herd alarm threshold were evaluated for their association with herd size and calving pattern.

Respondents were classified by herd size using information from the Teagasc advisory and ICBF databases, categories were defined based on the Irish national dairy herd average size (93 cows; [16]) as large (>150 cows), above average (100 – 150 cows), average (60 – 100 cows), or small (<60 cows); herd size information was obtained for 510 of the respondents.

Respondents were also classified by calving pattern using the information provided in the survey (spring-calving only, autumn-calving only, split-calving, or all year round calving) with only the two most commonly reported calving patterns being used in analysis (spring- and split-calving). Further herd-level descriptive information (305-day milk yield and calving interval) was obtained from the ICBF database.

Summary statistics were produced using the MEANS and FREQ procedures of SAS (Version 9.4; SAS Institute Inc., Cary, NC). Univariate logistic regression models were used to evaluate the association between reported disease treatment incidence and implemented management strategies with herd size or calving pattern using the GENMOD procedure of SAS. Statistical models included the logit link function and the Tukey-Kramer adjustment to account for multiple pairwise comparisons (herd size models). And the association between reported disease treatment incidence and herd size or calving pattern was only evaluated if the proportion of respondents above or below the respective herd alarm level was at least 20%. Reported odds ratio (OR) represent the ratio for the odds of "yes" vs. "no" answer to each question for respondents belonging to different herd size or calving pattern categories, taking as a reference the most prevalent categories (large herd size and spring-calving). Only OR at $P \leq 0.05$ for the comparison are reported in the manuscript. Considering each respondent did not answer every question of the survey, the number of respondents per question (and answer) is provided as appropriate.

Results

A total of 601 survey responses were received between 28th September and 10th October (2022); yielding a survey response rate of 15.4%. Excluding the duplicated (n = 4) and the blank responses (n = 72), 525 responses were available for analyses. Geographical distribution

by county of survey respondents providing a valid Eircode is presented in Fig 1. On average, it took respondents 14 minutes to complete the survey. Responses are reported for all respondents (n = 525), by herd size (large: n = 154, above average: n = 134, average: n = 148, or small: n = 74) or calving pattern for the two most common calving systems (spring-calving: n = 439, or split-calving: n = 67). Denominator values are shown for each question and answer; lower denominator values indicate questions or answers skipped by some respondents.

Fig 1. Geographical distribution by county of survey respondents across the Republic of Ireland (n = 408 respondents with valid Eircodes).

Study population

Overall, respondents median herd size was 110 cows (interquartile range [IQR] = 78 – 162 cows) and mean herd size was 135 cows. Respondents mainly had spring-calving herds (84.3%; 439/521) whilst the remainder operated split-calving (12.9%; 67/521), all year round calving (2.3%; 12/521), or autumn-calving (0.6%; 3/521) herds. For farmers with an active ICBF account with relevant data available, mean 305-day milk yield was 6,857 L (IQR = 6,111 – 7,162 L; n = 237) and mean calving interval was 377 days (IQR = 367 – 381 days; n = 323) for 2022. Based on the amount of bought-in feed per cow per year, farmers classified themselves as high-input (>1 tonne of bought-in feed/cow; 51.6% [268/519]), low-input (≤1 tonne of bought-in feed/cow; 47.8% [248/519]), or zero-grazed grass fed all of the time (0.6%; 3/519). Herd descriptions by herd size and calving pattern are provided in S2 Table.

Disease incidence

Incidence of disease was reported to be highest in freshly calved cows (first 3 weeks after calving; 57.6%; 299/519) and in multiparous cows (51.9%; 266/513). Respondents reported that disease incidence was highest among cows calving at the end of the block calving season

(with late calvers; 48.0%; 245/510). However, a substantial cohort of respondents, indicated that problems arise during all of the calving season regardless of the stage (41.4%; 211/510) and that disease affects both, primiparous and multiparous cows (43.1%; 221/513; Fig 2). The complete distribution of reported disease incidence according to stage of calving season, stage of lactation and parity by herd size and calving pattern is presented in S3 Table. Overall most farmers reported to treat ≤3% of their herd for milk fever (77.0%; 401/521) and retained placenta (86.9%; 450/518), and <1% of their herd for grass tetany (82.6%; 419/507), ketosis (72.7%; 368/506), displaced abomasum and/or digestive problems (71.5%; 373/522), and metritis (52.4%; 263/502) on an 'average' year on their farm. Table 1 shows the complete distribution of reported proportion of cows treated by condition, herd size and calving pattern. The odds of farmers from split-calving herds reporting to treat >3% of the herd for milk fever were 1.8 times those of farmers from spring-calving herds (OR [95% CI] = 1.78 [1.02 – 3.12]; *P* = 0.042). The association between other diseases reported treatments incidence and herd size or calving pattern was not evaluated given the limited number of farmers reporting to treat a proportion of animals above the herd alarm levels described by Lean and DeGaris [17].

Fig 2. Reported distribution of highest disease incidence by cow parity (A; n = 513), stage of lactation (B; n = 520) and stage of calving season (C; n = 510) for all survey respondents.

Table 1. Reported proportion of respondents' herd treated for health conditions on an "average" year (% of respondents)

	Herd size[a]				Herd calving pattern[a]		
Condition and treated cows	Large	Above average	Average	Small	Spring-calving	Split-calving	All
Milk fever	n = 154	n = 133	n = 146	n = 74	n = 437	n = 67	n = 521
>10%	2.0	1.5	2.7	1.4	1.8	3.0	1.9
7 to 10%	4.6	5.3	2.1	1.4	3.7	4.5	3.8
4 to 6%	14.3	18.8	15.1	24.3	16.0	25.4	17.3

1 to 3%	55.2	48.9	48.0	33.8	49.0	43.3	48.4
<1%	24.0	25.6	32.2	39.2	29.5	23.9	28.6
Retained placenta	n = 154	n = 133	n = 146	n = 71	n = 435	n = 67	n = 518
>10%	0.0	1.5	0.0	0.0	0.5	0.0	0.4
7 to 10%	2.6	2.3	2.1	0.0	2.3	1.5	2.1
4 to 6%	11.0	10.5	13.7	5.6	10.1	13.4	10.6
1 to 3%	60.4	54.1	50.0	56.3	53.8	64.2	55.6
<1%	26.0	31.6	34.3	38.0	33.3	20.9	31.3
Metritis	n = 148	n = 127	n = 141	n = 72	n = 424	n = 63	n = 502
>10%	0.0	0.8	0.7	0.0	0.2	1.6	0.4
7 to 10%	2.0	1.6	2.8	1.4	2.4	0.0	2.0
4 to 6%	8.8	7.9	8.5	5.6	8.5	7.9	8.2
1 to 3%	45.3	39.4	29.8	29.2	34.9	47.6	37.1
<1%	43.9	50.4	58.2	63.9	54.0	42.9	52.4
Displaced abomasum	n = 153	n = 134	n = 147	n = 74	n = 438	n = 66	n = 522
>10%	0.0	0.8	0.0	0.0	0.2	0.0	0.2
7 to 10%	0.0	0.8	0.7	0.0	0.5	0.0	0.4
4 to 6%	1.3	1.5	1.4	4.1	1.8	1.5	1.7
1 to 3%	28.8	26.1	24.5	25.7	26.0	25.8	26.3
<1%	69.9	70.9	73.5	70.3	71.5	72.7	71.5
Grass tetany	n = 151	n = 131	n = 140	n = 71	n = 425	n = 65	n = 507
>10%	0.7	0.8	0.0	1.4	0.5	1.5	0.6
7 to 10%	1.3	0.0	0.0	0.0	0.2	1.5	0.4
4 to 6%	0.7	2.3	1.4	5.6	2.1	0.0	2.0
1 to 3%	11.9	13.0	16.4	15.5	14.6	10.8	14.4
<1%	85.4	84.0	82.1	77.5	82.6	86.2	82.6
Ketosis	n = 149	n = 130	n = 141	n = 72	n = 425	n = 66	n = 506
>10%	0.0	0.8	0.0	0.0	0.2	0.0	0.2
7 to 10%	0.7	0.0	0.0	0.0	0.0	1.5	0.2
4 to 6%	1.3	3.1	3.6	5.6	3.3	1.5	3.0
1 to 3%	25.5	20.0	25.5	25.0	22.6	33.3	23.9
<1%	72.5	76.2	70.9	69.4	73.9	63.6	72.7

[a]Herds were categorized by herd size (large: >150 cows, above average: 100-150 cows, average: 60-100 cows, or small: <60 cows) based on the Irish national dairy herd average size (93 cows; [16]), and by calving pattern (spring-calving: cows calving in spring, or split-calving: cows calving in spring and autumn).

Perceived disease importance

Based on incidence and impact in their herd, most of the respondents indicated that occasional cases without major effect on herd performance were observed for milk fever and/or downer cow (73.0%; 381/522), metritis (72.2%; 374/518), ketosis (70.0%; 319/523), retained placenta (69.1%; 357/517), and displaced abomasum and/or digestive problems

(61.9%; 88/522). However, a noticeable proportion of the respondents indicated that milk fever was a significant (regularly treating severe cases with some cows lost/culled) or routine (regularly treating cows to control issues) problem in their herds (15.7%; 82/522). Subclinical hypocalcaemia was reported as a significant or routine problem in some herds (9.4%; 49/522), nevertheless, 20.7% (107/517) of farmers reported not knowing what subclinical hypocalcaemia was. The complete distribution of perceived disease importance as reported by herd size and calving pattern is described in S4 Table.

Disease records

Disease incidence records were kept by <55.0% of respondents for any of the evaluated conditions (Table 2). The odds of farmers from small herds reporting to keep records of metritis incidence were lower than those of farmers from large herds (OR [95% CI] = 0.35 [0.14 – 0.83]; P = 0.010), while odds of reporting incidence record keeping among farmers from different herd sizes and calving patterns were similar for other conditions (Table 3). Farmers frequently reported to keep records of antibiotic treatments for displaced abomasum and/or digestive problems (54.9%; 285/519), retained placenta (50.0%; 258/516), and metritis (39.4%; 199/505; Table 2). Additionally, some farmers, reported to keep records of antibiotic treatments for metabolic conditions [i.e. milk fever (23.7%; 123/518), ketosis (23.5%; 117/498) and grass tetany (15.5%; 79/509); Table 2]. The odds of farmers from small herds reporting to keep records of antibiotic treatments for milk fever were 3 times those of farmers from large herds (OR [95% CI] = 3.2 [1.42 – 7.26]; P < 0.001), while odds were similar for farmers from average and above average compared to those of farmers from large herds (Table 3).

Table 2. Reported dairy cow peripartum condition records kept (% of respondents)

	Herd size[a]				Herd calving pattern[a]		
Condition and record type	Large	Above average	Average	Small	Spring-calving	Split-calving	All
Milk fever	n = 153	n = 131	n = 145	n = 74	n = 430	n = 66	n = 518
Antibiotic treatment	17.6	24.4	22.1	40.5	25.1	18.2	23.7
Supportive treatment	30.7	29.0	35.9	35.1	33.5	27.3	32.6
Incidence	41.2	42.7	42.8	35.1	43.7	34.8	41.9
Retained placenta	n = 149	n = 133	n = 146	n = 73	n = 430	n = 65	n = 516
Antibiotic treatment	48.3	48.1	55.5	46.6	49.5	55.4	50.0
Supportive treatment	21.5	18.8	22.6	23.3	21.4	23.1	21.3
Incidence	49.7	49.6	43.8	32.9	47.9	43.1	45.9
Metritis	n = 148	n = 130	n = 141	n = 72	n = 422	n = 64	n = 505
Antibiotic treatment	39.2	39.2	39.0	40.3	39.1	45.3	39.4
Supportive treatment	14.9	13.8	13.5	13.9	13.0	17.2	13.9
Incidence	41.9	30.8	31.2	19.4	35.5	28.1	33.7
Displaced abomasum	n = 152	n = 133	n = 145	n = 74	n = 431	n = 66	n = 519
Antibiotic treatment	58.6	53.4	53.1	55.4	55.2	57.6	54.9
Supportive treatment	9.9	7.5	12.4	10.8	10.0	10.6	10.0
Incidence	36.8	33.8	35.2	31.1	36.2	34.8	35.1
Grass tetany	n = 148	n = 131	n = 145	n = 71	n = 423	n = 65	n = 509
Antibiotic treatment	12.2	14.5	17.2	21.1	15.4	15.4	15.5
Supportive treatment	19.6	21.4	20.0	14.1	20.6	15.4	19.6
Incidence	31.8	34.4	28.3	21.1	32.2	21.5	30.3
Ketosis	n = 150	n = 124	n = 134	n = 73	n = 414	n = 64	n = 498
Antibiotic treatment	22.7	22.6	22.4	31.5	22.9	26.6	23.5
Supportive treatment	18.7	15.3	16.4	13.7	15.5	18.8	16.3
Incidence	26.7	25.0	27.6	24.7	27.8	20.3	26.5

[a]Herds were categorized by herd size (large: >150 cows, above average: 100-150 cows, average: 60-100 cows, or small: <60 cows) based on the Irish national dairy herd average size (93 cows; [16]), and by calving pattern (spring-calving: cows calving in spring, or split-calving: cows calving in spring and autumn).

Table 3. Herd size odds ratios and 95% CI for responses to survey questions

Survey question and answer[a]	Class contrast (Herd size/calving pattern)[a]	Odds ratio (95% CI)[b]	*P*-value[c]
Management strategy			
Management in >1 group	Small vs. Large	0.22 (0.10, 0.50)	<0.001
	Average vs. Large	0.73 (0.40, 1.33)	0.537
	Above average vs. Large	0.64 (0.40, 1.03)	0.249
Management in >1 group	Split- vs. spring-calving	0.51 (0.30, 0.86)	0.011
Provide feed sources except silage	Split- vs. spring-calving	2.48 (1.46, 4.24)	<0.001
Once-a-day milking after calving	Split- vs. spring-calving	0.16 (0.07, 0.38)	<0.001
Cows indoors for a period after calving	Split- vs. spring-calving	0.34 (0.20, 0.57)	<0.001
Disease treatment incidence			
Milk fever	Split- vs. spring-calving	1.78 (1.02, 3.12)	0.042
Record type			
Metritis incidence	Small vs. Large	0.35 (0.14, 0.83)	0.010
	Average vs. Large	0.63 (0.34, 1.17)	0.223
	Above average vs. Large	0.63 (0.33, 1.20)	0.256
Antibiotic usage for milk fever	Small vs. Large	3.20 (1.42, 7.26)	<0.001
	Average vs. Large	1.30 (0.61, 2.74)	0.808
	Above average vs. Large	1.48 (0.69, 3.13)	0.546

[a]Herds were categorized by herd size (large: >150 cows, above average: 100-150 cows, average: 60-100 cows, or small: <60 cows) based on the Irish national dairy herd average size (93 cows; [14]), and by calving pattern (spring-calving: cows calving in spring, or split-calving: cows calving in spring and autumn).

[b]Contrast analysed as "yes" vs. "no" except for milk fever reported treatment incidence (≤3% or >3%).

[c]Values were adjusted using the Tukey-Kramer adjustment for multiple comparisons in the herd size model.

Dry cow management

Most commonly implemented management strategies for dry cows were body condition monitoring (73.4%; 365/497) and Mg and/or dry cow mineral supplementation in diet (61.2%; 304/497; Fig 3). The least reported management strategies were feeding a low K diet (20.3%; 101/497) or an acidifying diet [dietary cation-anion difference (DCAD); 6.2%; 31/497; Fig 3]. Some differences on reportedly implemented management strategies by herd size and calving pattern were observed (Table 3; Fig 3). Managing dry cows in more than one group (e.g. separate groups for fat and thin cows) was less frequently reported by farmers from small than large herds (OR [95% CI] = 0.22 [0.10 – 0.50]; $P < 0.001$) and by farmers from split- than spring-calving herds (OR [95% CI] = 0.51 [0.30 – 0.86]; $P = 0.011$; Table 3). The odds of farmers from split-calving herds reporting to provide feeds other than silage to dry cows were 2.5 times those of farmers from spring-calving herds (OR [95% CI] = 2.48 [1.46 – 4.24]; $P < 0.001$; Table 3). The full distribution of reported management strategies by herd size and calving pattern is presented in S5 Table.

Fig 3. Dry cows reportedly implemented management strategies by herd size [A; large (>150 cows; n = 148), above average (100-150 cows; n = 129), average (60-100 cows; n = 142) and small (<60 cows; n = 72)] and herd calving pattern [B; spring-calving (n = 428) and split-calving (n = 67)].

Fresh cow management

The most commonly implemented fresh cow management strategy in relation to transition cow disease prevention was Ca supplementation at calving (60.6%; 314/487; Fig 4); of these, 82.2% (258/314) reported to supplement only “high-risk” cows and 12.1% (38/314) reported to supplement all cows (18 respondents chose both options). Some differences in

implemented management strategies by herd size and calving pattern were observed (Table 3; Fig 4). Milking cows once-a-day for a period after calving was less frequently reported to be implemented by farmers from split- calving than from spring-calving herds (OR [95% CI] = 0.16 [0.07 – 0.38]; $P < 0.001$). Last, keeping freshly calved cows indoors for a period after calving (the overall most frequently reported management strategy; 68.0%; 331/487) was less frequently reported by farmers from split- than spring-calving herds (OR [95% CI] = 0.34 [0.20 – 0.57]; $P < 0.001$). The full distribution of reported management strategies by herd size and calving pattern is presented in S6 Table.

Fig 4. Fresh cows reportedly implemented management strategies by herd size [A: large (>150 cows; n = 148), above average (100-150 cows; n = 121), average (60-100 cows; n = 136) and small (<60 cows; n = 67) and herd calving pattern [B: spring-calving (n = 416) and split-calving (n = 67)

Discussion

A final total of 525 responses were suitable for data analysis, this represents 3.4% of Irish dairy herds (total of 15,319 dairy herds in 2022; [16]). Overall, respondents to this survey had larger herds and above average performance when compared to national averages; respondents mean herd size was 45% higher than the mean dairy herd size in the Republic of Ireland which is 93 cows [14], respondents mean 305-day milk yield and calving interval were respectively 20% higher and 3% lower than the 2022 national means (5,716 L/cow; [16]) and 388 days (ICBF HerdPlus users; [16]). The apparent 'above average' profile of the respondents' herds is not surprising as this survey was distributed among Teagasc Technical Dairy Advisory clients which tend to operate at a higher standard of technical and financial performance than the overall dairy farmer population in Ireland [18]. It has to be noted that farmers chose to

fill in the survey, thus further potential bias exists in the sample population as farmers interested in, or who are experiencing some issues with transition cow health and management may have been more likely to answer this survey. Respondents mostly had spring-calving herds which are most commonly seen in Ireland given the seasonal grass growth (92% of dairy herds; [19]) and the majority of respondents were located in county Cork (36.0%; 147/408; Fig 1), which is the county with the highest number of dairy cows in Ireland [20].

Regarding the reported disease levels, a herd alarm milk fever incidence threshold of >3% (within 14 days post-calving) was described by Lean and DeGaris [17] in an Australian technical review using data from grazing and confined herds; based on this threshold, 23.0% of respondents to our survey should be seeking help in regards to milk fever prevention. The provided threshold for retained placenta in this same review (>12 hours after calving; >6%) suggests that 2.5% of respondents to our survey should be seeking help for this condition if their definition of retained placenta aligned with the one used in the review. While research on subclinical hypocalcaemia in grazing cows is limited, and a study by Hendriks et al. [24] did not detect associations between subclinical hypocalcaemia and milk yield, milk solids, body condition score (BCS), blood non-esterified fatty acids or β-hydroxybutyrate (BHB) concentrations in grazing cows; subclinical hypocalcaemia is a recurrent topic of research worldwide [22] suggesting that transfer (or uptake) of scientific outputs to Irish dairy farmers may be limited (21% reported to do not know what subclinical hypocalcaemia was).

Given the low number of farmers reporting to keep disease records, the creation and promotion of strategies to improve record-keeping on farms should be an area of focus for outreach activities. Disease incidence and treatment record-keeping is paramount in

identifying patterns of disease and in aiding management of a disease at herd-level [23]. Our results also suggest that inappropriate antibiotic treatment decisions for metabolic disease treatment may be made at the farm-level. In the context of confined cows where extra-label use of antibiotics in the peripartum has been described, training the farmworkers involved in administering treatments to sick cows has proved successful at increasing their knowledge on transition cow disease diagnosis and treatment, without succeeding at decreasing overall antimicrobial use on farm [24,25].

In terms of dry cow management strategies, the importance of optimizing body condition at calving for subsequent health and reproductive performance and Mg supplementation to reduce the risk of milk fever in grazing systems has been emphasized for decades, thus, it is not surprising that the message has reached Irish dairy farmers and these are commonly reported dry cow management strategies [26–28]. In agreement with our findings, an Irish survey by Cummins et al. [15] reported that most of the respondents to their survey (n = 262) set a target calving BCS and fed dry cow minerals. Managing cows in >1 group during the dry period was one of the most commonly reported management strategies for this period, grouping cows by BCS is recommended for optimal BCS management during the dry period and BCS monitoring was the most commonly reported strategy in this study, however, we did not enquire about the management associated with the grouping strategy.

Low K diets are recommended for transition cows given K's contribution to a positive DCAD ultimately interfering with calcium metabolism and its impairment of dietary Mg absorption, and negative DCAD diets have solidly proven successful for milk fever prevention in confined cows [29,30]. The high K concentration and DCAD in pasture have been described as limiting factors for the implementation of these strategies in grazing systems [8]. Research in grazing

cows reports no association between positive DCAD (350 to 535 mEq/kg DM) and high K concentration (3.3 to 4.2% of DM) in pasture and plasma Ca concentration at calving, suggesting that these factors may not be key determinants of milk fever risk in grazing cows [8,31]. Nevertheless, K concentrations in Irish grass silage may not be as high as those reported from pasture in New Zealand's studies (mean [range] = 2.4% [0.6 to 5.6%] of DM; n = 1,636 samples; [32]); and thus opting for a low K grass silage or achieving a lower DCAD through the addition of anionic salts may be management strategies more suitable for dry cow feeding in the Irish dairy production system than in other grazing systems. Therefore, further research is needed to understand the limited uptake and to identify the barriers for the adoption and implementation of these research-supported strategies for milk fever prevention by Irish dairy farmers.

The most reported fresh cow strategy was keeping cows indoors for a period postpartum, a practice more commonly implemented in spring-calving dairy herds. This strategy is most likely implemented due to excessive soil moisture during the first months of the spring calving season (January and February; [33]) rather than by a transition cow health improvement desire. Split-calving herds use a lower amount of grazed grass in their cows diet potentially explaining the lower implementation among these farmers [34]. Calcium supplementation at calving was the next most commonly reported strategy that could be associated with a transition cow health improvement desire; this practice is regarded as a prophylactic strategy for hypocalcaemia, effective at temporarily increasing blood Ca concentration and leading to positive performance effects on subpopulations of animals [35]. Within this survey question, answers of supplementing "high-risk cows" and supplementing "all cows" at calving were combined; we did not ask farmers to outline their definition of a "high-risk" cow or their supplementation protocol, both of which are paramount in reaping the benefits of this

management strategy according to research conducted in confined cows. To the best of our knowledge, only two studies by the same authors have evaluated Ca supplementation at calving, choosing an oral form, in commercial Irish dairy farms; the first one (n = 91 cows) reporting a decrease in milk BHB concentration at days 14 and 28 post-partum and 1.3 kg/d higher milk yields up to 90 days in milk for supplemented multiparous cows, and the second one (n = 103 cows) reporting a decrease in milk BHB concentration at days 14 and 21 post-partum but no effect in production performance for supplemented multiparous cows [36,37]. Further research evaluating Ca supplementation strategies in the Irish dairy production context is warranted to optimise this commonly implemented strategy. Once-a-day milking was the third most popularly reported fresh period management strategy, this practice enables labour savings [38], and may reduce metabolite and mineral imbalances in early lactation and improve return to cyclicity after calving [39–41]; nevertheless our study did not enquire about the reasons behind the reported management strategies.

Conclusions

Results from the present study suggest that milk fever is a transition cow health concern in Irish dairy farms. Optimization of commonly implemented dry cow (Mg and/or dry cow mineral supplementation) and fresh cow (Ca supplementation at calving) management strategies, as well as enhanced uptake of dry cow management strategies proven successful under other production systems (low K and negative DCAD diet) may help reduce milk fever's burden on Irish dairy farms. Further research should identify the factors limiting the effectiveness of implemented management strategies and the end user adoption of successful management strategies for milk fever prevention. Additionally, dissemination activities targeting farmers from all herd sizes would be beneficial to increase awareness of

peripartum metabolic diseases and their recommended treatment, as well as to promote disease incidence and treatment record keeping.

Acknowledgements

The authors are especially thankful to the farmers who responded to this survey. And to J. Mason (Teagasc, Animal and Grassland Research and Innovation Centre, Moorepark, Fermoy, Ireland) for her work compiling herd-level information.

References

1. Roche JR, Bell AW, Overton TR, Loor JJ. Nutritional management of the transition cow in the 21st century–a paradigm shift in thinking. Anim Prod Sci. 2013;53: 1000–1023. doi:10.1071/AN12293.

2. Kay JK, Loor JJ, Heiser A, McGowan J, Roche JR. Managing the grazing dairy cow through the transition period: a review. Anim Prod Sci. 2015;55: 936. doi:10.1071/AN14870.

3. Mulligan FJ, Doherty ML. Production diseases of the transition cow. Vet J. 2008;176: 3–9.

4. Spaans OK, Kuhn-Sherlock B, Hickey A, Crookenden MA, Heiser A, Burke CR, et al. Temporal profiles describing markers of inflammation and metabolism during the transition period of pasture-based, seasonal-calving dairy cows. J Dairy Sci. 2022;105: 2669–2698.

5. Lange J, McCarthy A, Kay J, Meier S, Walker C, Crookenden MA, et al. Prepartum feeding level and body condition score affect immunological performance in grazing dairy cows during the transition period. J Dairy Sci. 2016;99: 2329–2338. doi:

10.3168/jds.2015-10135.

6. Roche JR, Meier S, Heiser A, Mitchell MD, Walker CG, Crookenden MA, et al. Effects of precalving body condition score and prepartum feeding level on production, reproduction, and health parameters in pasture-based transition dairy cows. J Dairy Sci. 2015;98: 7164–7182. doi:10.3168/jds.2014-9269.

7. Mills KE, Weary DM, von Keyserlingk MAG. Identifying barriers to successful dairy cow transition management. J Dairy Sci. 2020;103: 1749–1758. doi:10.3168/jds.2018-16231.

8. Roche J. Transition Management in Grazing Systems: Pragmatism Before Precision. Vet Clin North Am Food Anim Pract. 2023;39: 325–336. doi:10.1016/j.cvfa.2023.02.005.

9. Aubé L, Mialon MM, Mollaret E, Mounier L, Veissier I, de Boyer des Roches A. Review: Assessment of dairy cow welfare at pasture: measures available, gaps to address, and pathways to development of ad-hoc protocols. Animal. 2022;16: 100597. doi:10.1016/j.animal.2022.100597.

10. United States Department of Agriculture. National Animal Health Monitoring System (NAHMS). 2014. Dairy cattle management practices in the United States. 2014.

11. Daros RR, Hötzel MJ, Bran JA, LeBlanc SJ, von Keyserlingk MAG. Prevalence and risk factors for transition period diseases in grazing dairy cows in Brazil. Prev Vet Med. 2017;145: 16–22. doi:10.1016/j.prevetmed.2017.06.004.

12. Fujiwara M, Haskell MJ, Macrae AI, Rutherford KMD. Survey of dry cow management on UK commercial dairy farms. Vet Rec. 2018;183: 297. doi:10.1136/vr.104755.

13. Cummins C, Berry DP, Sayers R, Lorenz I, Kennedy E. Questionnaire identifying

management practices surrounding calving on spring-calving dairy farms and their associations with herd size and herd expansion. Animal. 2016;10: 868–877. doi:10.1017/S1751731116000124.

14. Dillon E, Donnellan T, Moran B, Lennon J. Teagasc National Farm Survey 2022 Preliminary Results. 2023 [cited 10 Dec 2023]. Available from: https://www.teagasc.ie/media/website/publications/2023/NFS_prelim_results_2022.pdf.

15. Xu Y, Pace S, Kim J, Iachini A, King LB, Harrison T, et al. Threats to online surveys: Recognizing, detecting, and preventing survey bots. Soc Work Res. 2022;46: 343–350. doi:10.1093/swr/svac023.

16. ICBF. HerdPlus Dairy Calving Statistics 2022. 2022 [cited 13 May 2024]. Available from: https://www.icbf.com/herdplus-dairy-calving-statistics-2022/.

17. Lean I, DeGaris P. Transition cow management a technical review for nutritional professionals, veterinarians and farm advisers. 2021. [cited 10 Dec 2023]. Available from: https://www.researchgate.net/publication/350850407_transition_cow_management_a_technical_review_for_nutritional_professionals_veterinarians_and_farm_advisers_transition_period_mating_mid-late_lactation_Dry_period_Calving_Publisher_Dairy_Australia.

18. Bogue P. Impact of participation in Teagasc dairy discussion groups. 2013 [cited 1 Mar 2024]. Available from: https://www.teagasc.ie/media/website/publications/2013/Discussion_Group_Report

_Web_Jan2013.pdf.

19. National Milk Agency. National Milk Agency Annual Report & Accounts 2022. 2022 [cited 1 Mar 2023]. Available from: https://nationalmilkagency.ie/wp-content/uploads/2023/07/NMA-Annual-Report-2022.pdf.

20. Central Statistics Office. Dairy Farming. 2020 [cited 2 Nov 2023]. Available from: https://www.cso.ie/en/releasesandpublications/ep/p-syi/statisticalyearbookofireland2021part3/agri/dairyfarming/.

21. Hendriks SJ, Huzzey JM, Kuhn-Sherlock B, Turner S-A, Mueller KR, Phyn CVC, et al. Associations between lying behavior and activity and hypocalcemia in grazing dairy cows during the transition period. J Dairy Sci. 2020;103: 10530–10546. doi:10.3168/jds.2019-18111.

22. Couto Serrenho R, DeVries TJ, Duffield TF, LeBlanc SJ. Graduate Student Literature Review: What do we know about the effects of clinical and subclinical hypocalcemia on health and performance of dairy cows? J Dairy Sci. 2021;104: 6304–6326. doi:10.3168/jds.2020-19371.

23. Rhoda DA, Pantoja JCF. Using mastitis records and somatic cell count data. Vet Clin North Am Food Anim Pract. 2012;28: 347–361. doi:10.1016/j.cvfa.2012.03.012.

24. Silva-del-Río N, Valldecabres A, Espadamala A, García-Muñoz A, Pallares P, Lago A, et al. Treatment practices after calving-related events on 45 dairy farms in California. J Dairy Sci. 2021;104: 12164–12172. doi:10.3168/jds.2021-20593.

25. Garzon A, Portillo R, Habing G, Silva-del-Rio N, Karle BM, Pereira R V. Antimicrobial stewardship on the dairy: Evaluating an on-farm framework for training farmworkers.

J Dairy Sci. 2023;106: 4171–4183. doi: 10.3168/jds.2022-22560.

26. Roche JR, Macdonald KA, Burke CR, Lee JM, Berry DP. Associations among body condition score, body weight, and reproductive performance in seasonal-calving dairy cattle. J Dairy Sci. 2007;90: 376–391. doi:10.3168/jds.S0022-0302(07)72639-5.

27. Roche JR, Kay JK, Friggens NC, Loor JJ, Berry DP. Assessing and managing body condition score for the prevention of metabolic disease in dairy cows. Vet Clin North Am - Food Anim Pract. 2013;29: 323–336. doi:10.1016/j.cvfa.2013.03.003.

28. Lean IJ, DeGaris PJ, McNeil DM, Block E. Hypocalcemia in Dairy Cows: Meta-analysis and Dietary Cation Anion Difference Theory Revisited. J Dairy Sci. 2006;89: 669–684. doi:10.3168/jds.S0022-0302(06)72130-0.

29. National Academies of Sciences, Engineering and Medicine. Energy. In: Nutrient Requirements of Dairy Cattle. Eighth Revised Edition. Washington, DC: The National Academies Press; 2021. p. 32.

30. Santos JEP, Lean IJ, Golder H, Block E. Meta-analysis of the effects of prepartum dietary cation-anion difference on performance and health of dairy cows. J Dairy Sci. 2019;102: 2134–2154. doi:10.3168/jds.2018-14628.

31. Roche JR, Morton J, Kolver ES. Sulfur and Chlorine Play a Non-Acid Base Role in Periparturient Calcium Homeostasis. J Dairy Sci. 2002;85: 3444–3453. doi: 10.3168/jds.S0022-0302(02)74432-9.

32. Rogers P, Murphy W. Levels of dry matter, major elements (calcium, magnesium, nitrogen, phosphorus, potassium, sodium and sulphur) and trace elements (cobalt, copper, iodine, manganese, molybdenum, selenium and zinc) in Irish grass, silage and

hay. 2000 [cited 27 Feb 2024]. Available from: http://homepage.tinet.ie/~progers/0forage.htm.

33. Schulte RPO, Diamond J, Finkele K, Holden NM, Brereton AJ. Predicting the soil moisture conditions of Irish grasslands. Irish J Agric Food Res. 2005;44: 95–110. doi: 10.1017/S0021859613000397.

34. Geary U, Lopez-Villalobos N, Garrick DJ, Shalloo L. Spring calving versus split calving: effects on farm, processor and industry profitability for the Irish dairy industry. J Agric Sci. 2014;152: 448–463. doi:10.1017/S0021859613000397.

35. Valldecabres A, Branco-Lopes R, Bernal-Córdoba C, Silva-del-Río N. Production and reproduction responses for dairy cattle supplemented with oral calcium bolus after calving: Systematic review and meta-analysis. JDS Commun. 2023;4: 9–13. doi:10.3168/jdsc.2022-0235.

36. Lawlor J, Fahey A, Neville E, Stack A, Mulligan F. Effect of Cow Start Calcium Bolus on Metabolic Status and Milk Production in Early Lactation. Anim Vet Sci. 2020;8: 124. doi:10.11648/j.avs.20200806.12.

37. Lawlor J, Fahey A, Neville E, Stack A, Mulligan F. On-farm Safety and Efficacy Trial of Cow Start Calcium Bolus. Anim Vet Sci. 2019;7: 121–126. doi:10.11648/j.avs.20190706.11.

38. Murphy JP, O'Donovan M, McCarthy K, Delaby L, Sugrue K, Galvin N, et al. A three-year comparison of once-a-day and twice-a-day milking in seasonal-calving pasture-based systems. J Dairy Sci. 2023;106: 8910–8925. doi:10.3168/jds.2023-23379.

39. Patton J, Kenny DA, Mee JF, O'Mara FP, Wathes DC, Cook M, et al. Effect of milking

frequency and diet on milk production, energy balance, and reproduction in dairy cows. J Dairy Sci. 2006;89: 1478–1487. doi:10.3168/jds.S0022-0302(06)72215-9.

40. Loiselle MC, Ster C, Talbot BG, Zhao X, Wagner GF, Boisclair YR, et al. Impact of postpartum milking frequency on the immune system and the blood metabolite concentration of dairy cows. J Dairy Sci. 2009;92: 1900–1912. doi:10.3168/jds.2008-1399.

41. Valldecabres A, Lopes RB, Lago A, Blanc C, Silva-del-Río N. Effects of postpartum milking strategy on plasma mineral concentrations and colostrum, transition milk, and milk yield and composition in multiparous dairy cows. J Dairy Sci. 2022;105: 595–608. doi:10.3168/jds.2021-20590.

Supporting information

S1 Table. Transition cow health and management questions sent in a survey via text message to 3 899 Teagasc (Agriculture and Food Development Authority in the Republic of Ireland) dairy advisory clients in October 2022. The survey was made using Survey Monkey (SurveyMonkey Inc., Palo Alto, CA) but has been presented here in table format.

S2 Table. Dairy cow herd descriptions for survey respondents with an active ICBF (Irish Cattle Breeding Federation) account by herd size and calving pattern. [a]Herds were categorized by herd size (large: >150 cows, above average: 100-150 cows, average: 60-100 cows, or small: <60 cows) using the Irish national dairy herd average as reference (93 cows; Dillon et al., 2023), and by calving pattern (spring-calving: cows calving in spring, or split-calving: cows calving in spring and autumn). [b]IQR = Interquartile range.

S3 Table. Reported highest observed disease incidence by cow parity, stage of lactation and stage of calving season presented by herd size and calving pattern (% of respondents). [a]Herds were categorized by herd size (large: >150 cows, above average: 100-150 cows, average: 60-100 cows, or small: <60 cows) using the Irish national dairy herd average as reference (93 cows; Dillon et al., 2023), and by calving pattern (spring-calving: cows calving in spring, or split-calving: cows calving in spring and autumn). [b]Stages of lactation: Fresh calver: First 3 weeks after calving, early lactation: from week 3 to end of 3rd month of lactation, mid lactation: from start of 4th month to end of 7th month of lactation, late lactation: from start of 8th month of lactation to dry-off, far-off dry: from dry-off to close-up, close-up dry: last 3 weeks of pregnancy.

S4 Table. Reported perception of dairy cow diseases by herd size and calving pattern. Perception was based on treatments, mortality, culling and herd performance (% of respondents). [a]Herds were categorized by herd size (large: >150 cows, above average: 100-150 cows, average: 60-100 cows, or small: <60 cows) using the Irish national dairy herd average as reference (93 cows; Dillon et al., 2023), and by calving pattern (spring-calving: cows calving in spring, or split-calving: cows calving in spring and autumn). [b]Perception definitions: Significant problem (regularly treating severe cases with some cows lost/culled), routine problem (regularly treating cows to control issues), occasional cases (but no major effect on herd performance)

S5 Table. Reported dry cow management strategies by herd size and calving pattern (% of respondents). [a]Herds were categorized by herd size (large: >150 cows, above average: 100-150 cows, average: 60-100 cows, or small: <60 cows) using the Irish national dairy herd average as reference (93 cows; Dillon et al., 2023), and by calving pattern (spring-calving: cows calving in spring, or split-calving: cows calving in spring and autumn). [b]DCAD = Dietary cation anion difference.

S6 Table. Reported fresh cow management strategies by herd size and calving pattern (% of respondents). [a]Herds were categorized by herd size (large: >150 cows, above average: 100-150 cows, average: 60-100 cows, or small: <60 cows) using the Irish national dairy herd average as reference (93 cows; Dillon et al., 2023), and by calving pattern (spring-calving: cows calving in spring, or split-calving: cows calving in spring and autumn)

Figure 1

Click here to access/download;Figure;Fig1.tiff.tiff

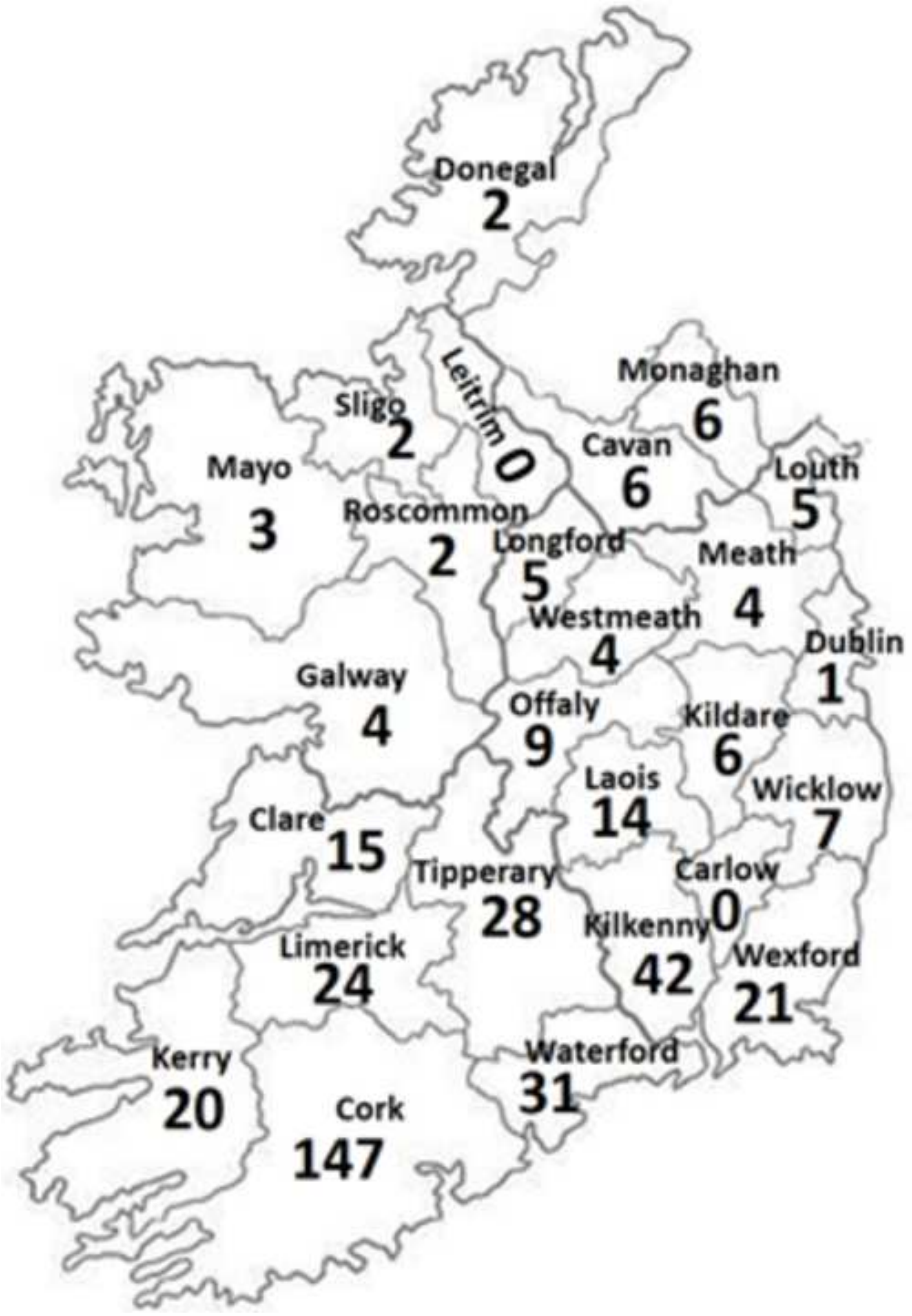

Figure 2

Click here to access/download;Figure;Fig2.tiff.tiff

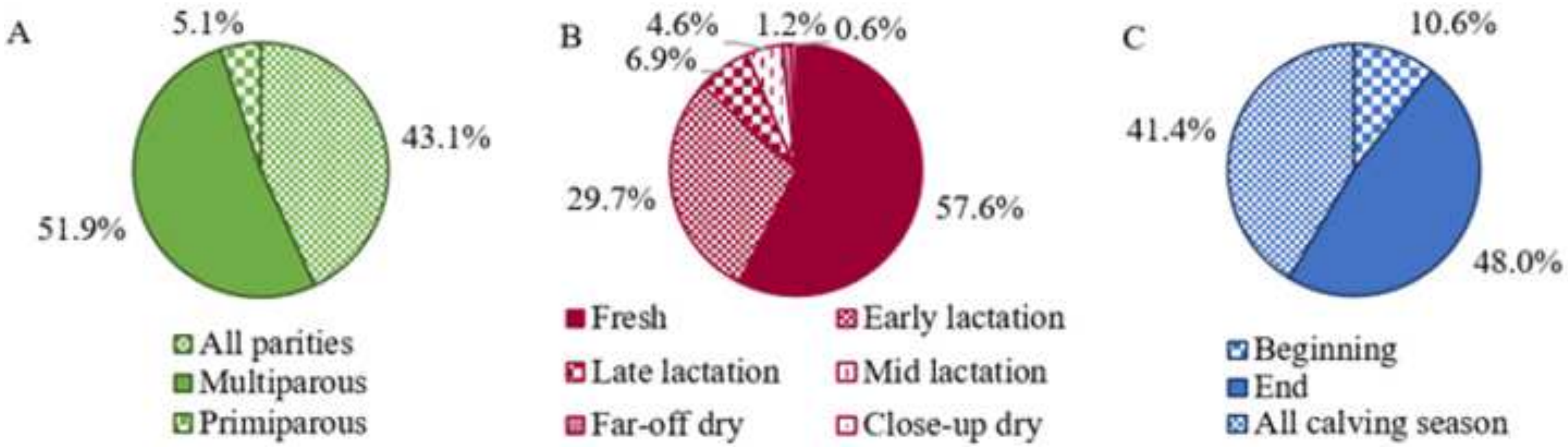

Figure 3

Click here to access/download;Figure;Fig3.tiff.tiff

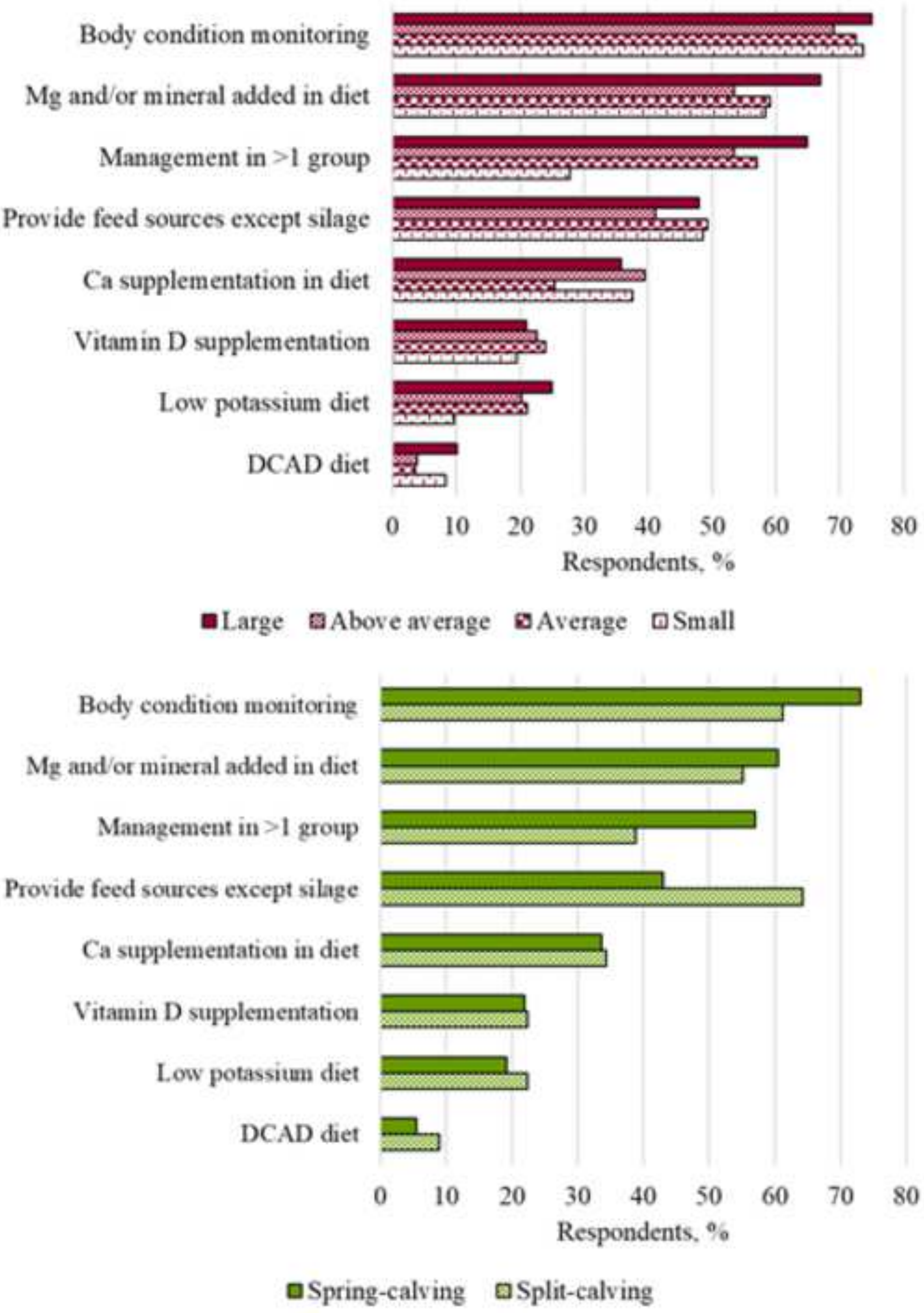

Figure 4

Click here to access/download;Figure;Fig4.tiff.tiff

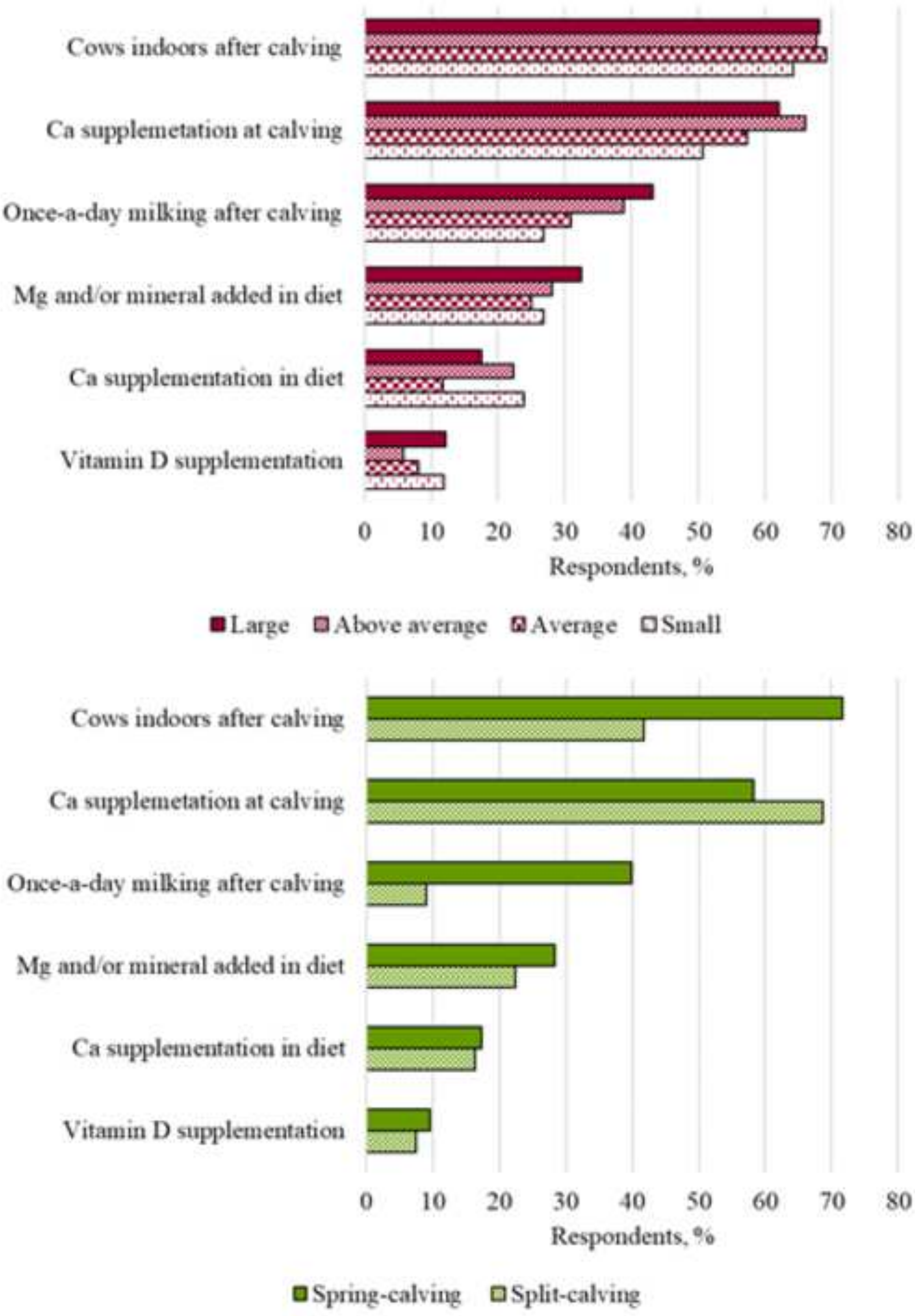

Click here to access/download
Supporting Information
S1_Table.docx.docx

Click here to access/download
Supporting Information
S2_Table.docx.docx

Click here to access/download
Supporting Information
S3_Table.docx.docx

Click here to access/download
Supporting Information
S4_Table.docx.docx

Supporting Information 5

Click here to access/download
Supporting Information
S5_Table.docx.docx

Click here to access/download
Supporting Information
S6_Table.docx.docx
